# Reorienting and rebuilding the health system in war-torn Tigray, Ethiopia

**DOI:** 10.1136/bmjgh-2021-007088

**Published:** 2021-08-27

**Authors:** Azeb Gebresilassie Tesema, Yohannes Kinfu

**Affiliations:** 1 Health System Sciences, The George Institute for Global Health, Newtown, New South Wales, Australia; 2 School of Public Health, College of Health Sciences, Mekelle University, Mekelle, Tigray, Ethiopia; 3 Faculty of Health, University of Canberra, Canberra, Australian Capital Territory, Australia; 4 Department of Health Metrics Sciences, University of Washington, Seattle, Washington, USA

**Keywords:** health systems

**Summary box**

- Conflict and war have direct and indirect effects on health and health systems.
- The war in the Tigray region of Ethiopia has already caused a considerable number of casualties, massive internal displacement and over 70 000 refugees in neighbouring Sudan. It also led to the collapse of the health system.
- The task of restoring and rebuilding Tigray’s health system requires maintaining a balance between providing urgent humanitarian assistance and building a resilient health system.
- It requires reorienting service delivery according to location and security level; redefining the essential health service package to meet new needs; building capacity for emergency response; reskilling and empowering the health workforce and coordinating actors and resources to create a sustainable and resilient health system.

## Introduction

Armed conflict and war, directly and indirectly, affect health and health systems.^1 2^ Globally, conflict was responsible for over 63 000 deaths and over 2.7 million years of lives lost due to disability in 2019.^3^ The war in the Tigray region of Ethiopia, which started in November 2020, has already caused a considerable number of casualties, massive internal displacement and over 70 000 refugees in neighbouring Sudan.^4^ A recent report by World Food Program suggests that 91% of the region’s six million people require immediate emergency humanitarian assistance, and 400 000 people had crossed the ‘threshold into famine’.^5 6^ Evidence also revealed that women and girls were subjected to sexual abuse and gender-based violence in this war.^6^ In addition, the destruction, vandalisation and looting of health facilities have left millions of people without access to essential healthcare.^7^

The process of restoring and rebuilding Tigray’s health system will likely be shaped by many factors and take several years. In this paper, we outline three interrelated action points that we think should guide any future effort: health system adaptation; protecting, reskilling and empowering the health workforce and coordinating actors and resources towards creating a sustainable and resilient health system. The priority areas are informed by Ethiopia’s own experience, available global frameworks and country-level evidence on conflict and health.^2 8–13^

## Health system adaptation

Before the war, the Tigray region had 47 hospitals, 224 health centres, 712 health posts and 269 functional ambulances. A recent assessment by United Nations Office for the Coordination of Humanitarian Affairs (UNOCHA) and others indicated that about 70% of assessed hospitals and health centres in the region were either partially or fully damaged during the war, most ambulances were either looted or destroyed and all the health posts were rendered non-functional.^14 15^ As a result, over 2.5 million people are without access to essential services. Part of this is due to a large number of internally displaced people (IDPs); an estimated one in three of the regional population—about 2 million people—are internally displaced.^14^

The presence of IDPs, the destruction of the health infrastructure and the volatile nature of the security situation in the region require health system adaptation to ensure that essential services are provided to the population. Specifically, it requires reorienting service delivery according to location and security level, building capacity for emergency response and rebuilding the system for resilience. In conflict epicentres, where violence is acute and ongoing, the priority should be on a small set of medically urgent life-saving interventions.^11^ In insecure areas, where there is no active conflict, but the threat of violence might be imminent, a wider range of preventive and curative interventions may be reintroduced in phases.^10 11^ These interventions can be initially offered through mobile clinics, satellite health posts and other outreach programmes. Where the security situation is better, ambulance services may support the interventions for severe cases, with a clear goal to build a better and resilient health system capable of providing core services and responding to man-made and other emergencies.

In areas of high population displacement, the redeployment of health extension workers (HEWs) and other healthcare workers within the IDP centres represent the best buy for the health system. And, there are many reasons for this. First, internal displacement usually generates new and high demand for health services which cannot be fully met through the work of humanitarian groups. Second, the HEWs themselves are most likely to be already within IDP centres, given the number of IDPs in the region. Third, HEWs are relatively abundant, have the trust of their community and are the backbone of the country’s health system. Fourth, they can meet and maintain ‘old’ service needs such as maternal and child health, HIV, Tuberculosis, malaria and non-communicable diseases services. Finally, engaging the HEWs in this process will help retain their existing skill; save loss of institutional memory and create opportunities for training on emergency response and preparedness. These efforts will have both shortand long-term benefits: it will reduce health worker attrition, cut the future cost of re-integration and help build a sustainable and resilient health system while responding to emerging and previously existing service needs, in which they are sufficiently trained.

While reinstating essential services, community involvement and mobilisation is critical, as shown in previous experience in Ethiopia.^8 12^ However, the exact set of services should be determined through a rapid but comprehensive needs assessment exercise, and it should be framed in the context of building a resilient health system that addresses emerging health burdens and the long-term effects of the war. The current Ethiopian essential health service package is not designed to handle such emergency service needs. Notably, the health extension programme does not include trauma management, mental healthcare, psychological support (including for victims of gender-based violence) and rehabilitation services. These require redefining the essential health service package and the core set of basic services within the health extension programme.

The rebuilding process should also go hand in hand with revitalising the health information system in the region. This is required because the existing paper-based system has been destroyed along with the health infrastructure. Even in the health centres and hospitals, including the woreda (district) office, where an electronic health information management system has been in place, the information may have been lost or rendered non-functional because of the looting and distraction of computers.^15^ The use of mobile health technology could help meet current needs and avert such future eventualities.

Furthermore, restoring and rebuilding health service delivery requires restocking medical equipment, drugs, vaccine and other medical supplies that have been destroyed or stocked out since the start of the war. Such effort should also include immediate restoration of basic infrastructures such as electricity, telephone system, ambulances and the road network as no health system is considered ready without them.

## Protecting, reskilling and empowering health workforce

Given the critical role of health workers in responding to emergencies and rebuilding the health system, securing a secure space for healthcare workers and protecting them from violence is paramount. Evidence from a systematic review based on papers mainly from Iraq, Israel, Palestine, Sierra Leone, Syria and Uganda suggests that health workers’ availability, motivation and performance can be directly affected by violence.^9 16^ Health workers can be victims or suffer from personal tragedies involving their immediate families.^10^ The most recent reports of the killings of Médecins Sans Frontières staff in the Tigray region vividly show the risks to health workers in the area.^17^

It is imperative for all health workers to feel safe. Protecting female health workers from violence and potential sexual and other abuse are critical to sustaining the health system. A global code of practice that affords the same level of protection for local health workers as those offered to international health staff and other humanitarian workers is urgently needed. Local and international players should work towards adopting an easily recognised unique uniform for local health workers to operate safely in emergencies.

Task shifting, reskilling and blended training are also required to meet the newly emerging health burdens and prepare health workers for comprehensive care needs. Furthermore, the scale and severity of the crisis, the extra workload resulting from increased needs but fewer responders, along with shortages of critical life-saving medication and equipment, could place further psychological distress and compromise health workers roles. Development partners need to invest in programmes that motivate, empower and help retain health workers, particularly in remote and hard-to-achieve areas, such as direct salary support and implementing flexible deployment strategies like those offered for international staff. In partnership with international organisations, the government could also consider secondment and voluntary service programmes to attract professionals, locally and internationally, to bridge the skill gap. However, such efforts require coordination and harmonisation to avoid pitfalls around incentives packages linked in part to the existence of multiple actors in postconflict settings.^13^

## Coordinating actors and resources for a sustainable health system

Conflict disrupts leadership and health system structures.^10 11^ A recent UN report revealed that the woreda level health administration structure in Tigray is non-existent or has limited function. For example, there is no system to monitor service delivery in the region.^14^ The displacement and disintegration of the existing health system are so much that the UN health cluster has been activated.^14^

Rebuilding health system governance in the region should draw from past experiences in Tigray and at the national level, which showed that success in postconflict recovery and long-term health system development requires coordination across players.^8 12^ There is a need to engage the community system, given the community’s vested interest and experience in dealing with a similar situation. Development partners should be subservient to local initiatives, focus on efficiency and work through existing health systems to ensure programme sustainability.^8 9^ Above all, the task of reconstruction needs to balance between providing urgent humanitarian assistance and working toward long-term redevelopment and building a resilient health system.^2 9^

## Conclusion

Standing where we are, it is difficult to predict what the future holds for Ethiopia, given the war which started in Tigray is now beginning to spread to the adjacent regions. However, considering health in the context of human welfare and the bigger picture, success in the political sphere is critical to avert complete chaos. Ethiopia’s own experiences on health and human development in the past three decades, after long-lasting conflict with severe health and infrastructure impacts, is a testimony to the dividend of peace and the feasibility of successfully emerging from a once ruined and fragile system.

## Data Availability

All data relevant to the study are included in the article.
